# Adenovirus Respiratory Tract Infections in Peru

**DOI:** 10.1371/journal.pone.0046898

**Published:** 2012-10-08

**Authors:** Julia S. Ampuero, Víctor Ocaña, Jorge Gómez, María E. Gamero, Josefina Garcia, Eric S. Halsey, V. Alberto Laguna-Torres

**Affiliations:** 1 U.S. Naval Medical Research Unit No. 6. Lima, Perú; 2 Centro de Salud Pachitea. Dirección Regional de Salud de Piura. Ministerio de Salud. Piura, Perú; 3 Dirección General de Epidemiología. Ministerio de Salud. Lima, Perú; University Hospital San Giovanni Battista di Torino, Italy

## Abstract

**Background:**

Currently, there is a paucity of data regarding human adenovirus (HAdv) circulation in Andean regions of South America. To address this shortcoming, we report the clinical, phylogenetic, and epidemiologic characteristics of HAdv respiratory tract infection from a large sentinel surveillance study conducted among adults and children in Peru.

**Methods/Principal Findings:**

Oropharyngeal swabs were collected from participants visiting any of 38 participating health centers, and viral pathogens were identified by immunofluorescence assay in cell culture. In addition, molecular characterization was performed on 226 randomly selected HAdv samples. Between 2000 and 2010, a total of 26,375 participants with influenza-like illness (ILI) or severe acute respiratory infection (SARI) were enrolled in the study. HAdv infection was identified in 2.5% of cases and represented 6.2% of all viral pathogens. Co-infection with a heterologous virus was found in 15.5% of HAdv cases. HAdv infection was largely confined to children under the age of 15, representing 88.6% of HAdv cases identified. No clinical characteristics were found to significantly distinguish HAdv infection from other respiratory viruses. Geographically, HAdv infections were more common in sites from the arid coastal regions than in the jungle or highland regions. Co-circulation of subgroups B and C was observed each year between 2006 and 2010, but no clear seasonal patterns of transmission were detected.

**Conclusions/Significance:**

HAdv accounted for a significant fraction of those presenting with ILI and SARI in Peru and tended to affect the younger population disproportionately. Longitudinal studies will help better characterize the clinical course of patients with HAdv in Peru, as well as determine the role of co-infections in the evolution of illness.

## Introduction

The *Adenoviridae* family has six subgroups (A to F) and 51 serotypes that infect humans [1], [2]. Various serotypes of human adenovirus (HAdv) may cause a wide spectrum of respiratory tract manifestations such as upper respiratory compromise, bronchiolitis, or pneumonia [3]. In addition, extrapulmonary manifestations, such as conjunctivitis, gastroenteritis, meningitis, encephalitis, acute hemorrhagic cystitis, or tubulointerstitial nephritis, may result from infection [4], [5], [6], [7]. The association of specific serotypes with a particular syndrome has not been fully elucidated although clinical manifestations are sometimes linked to the site of viral inoculation [8]. Underlying conditions (e.g., recent transplantation) and particular serotypes (e.g., 5 and 21) have been associated with increased adenoviral morbidity [9]. Subclinical or mild infections have also been described in as many as half of those infected [10] and are reasons why HAdv infection is often under-documented except in outbreaks. Furthermore, a carrier state may exist with carriage of the virus for as long as 906 days [11]; usual sites of persistence include the adenoids and lymphocytes, but the significance of this phenomenon is currently unknown [12].

The incubation period usually ranges from 4 to 8 days but has been demonstrated to be as long as 10 days [13], [14]. HAdv may be transmitted by a wide variety of routes including droplet [10], fomites (including improperly sterilized medical equipment and contaminated ophthalmic solution), fecal-oral, and auto-inoculation, including in healthcare settings [7], [15].

Community dwellings, such as military barracks, daycare centers, and nursing homes, have been associated with an increased risk for exposure, including outbreaks [16], [17], [18], [19]. Furthermore, inadequately chlorinated swimming pools and natural bodies of water have been linked to HAdv conjunctivitis outbreaks [20], [21].

In the United States, epidemiologic studies have shown that 1–5% of all respiratory infections are caused by HAdv [22]. This virus particularly causes problems among children younger than 5 years who may get 61% of all HAdv documented infections [23]. In addition to fever, patients with respiratory infection often present with pharyngitis, rhinorrhea, and cough [24]. Pharyngeal HAdv infections are virtually indistinguishable from bacterial infections and often possess features commonly associated with streptococcal pharyngitis, such as fever, tonsillar exudates (in as many as 52% of children), and leukocytosis [25]. In addition to the aforementioned respiratory symptoms, accompanying non-respiratory manifestations such as malaise, myalgias, conjunctivitis, and abdominal pain may also be observed.

In a respiratory disease passive surveillance network, our team has studied the etiology of viral infections prior to and during the influenza A/H1N1 pandemic, in Peru [26], [27]. In addition, we have determined that HAdv subgroups B, C, and E circulate within Peru and subgroup C predominated during the 2006–2008 period [28]. We also identified HAdv subgroup E in samples obtained from a conjunctivitis outbreak in Peruvian Navy personnel stationed in Lima, Peru (Virology Department, U.S. Naval Medical Research Unit No. 6 (NAMRU-6), unpublished data).

The aim of this study was to describe the clinical, phylogenetic, and epidemiologic characteristics of subjects with HAdv infections enrolled in a passive surveillance system for respiratory disease in Peru.

**Figure 1:**
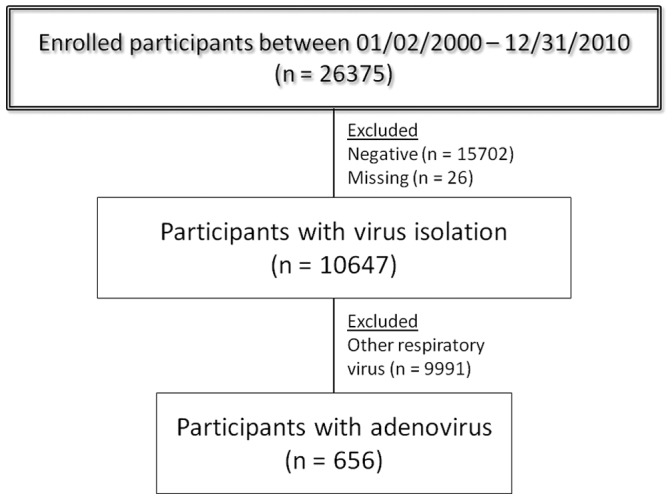
Flowchart for the analysis of patients with HAdv.

## Materials and Methods

### Study Population and Case Definition

The study population included every patient with influenza like illness (ILI) or with severe acute respiratory infection (SARI), regardless of age, who sought attention or was hospitalized in participating health facilities between January 2000 and December 2010, and agreed to participate. At each site, trained personnel were responsible for properly identifying and classifying patients with ILI or SARI. The case definition of ILI was a sudden onset of fever (oral temperature ≥38°C or axillary temperature ≥37.5°C) and either cough or sore throat. SARI included the ILI case definition plus dyspnea and the need for hospitalization [29]. All patients were recruited within five days of their initial symptoms.

**Figure 2:**
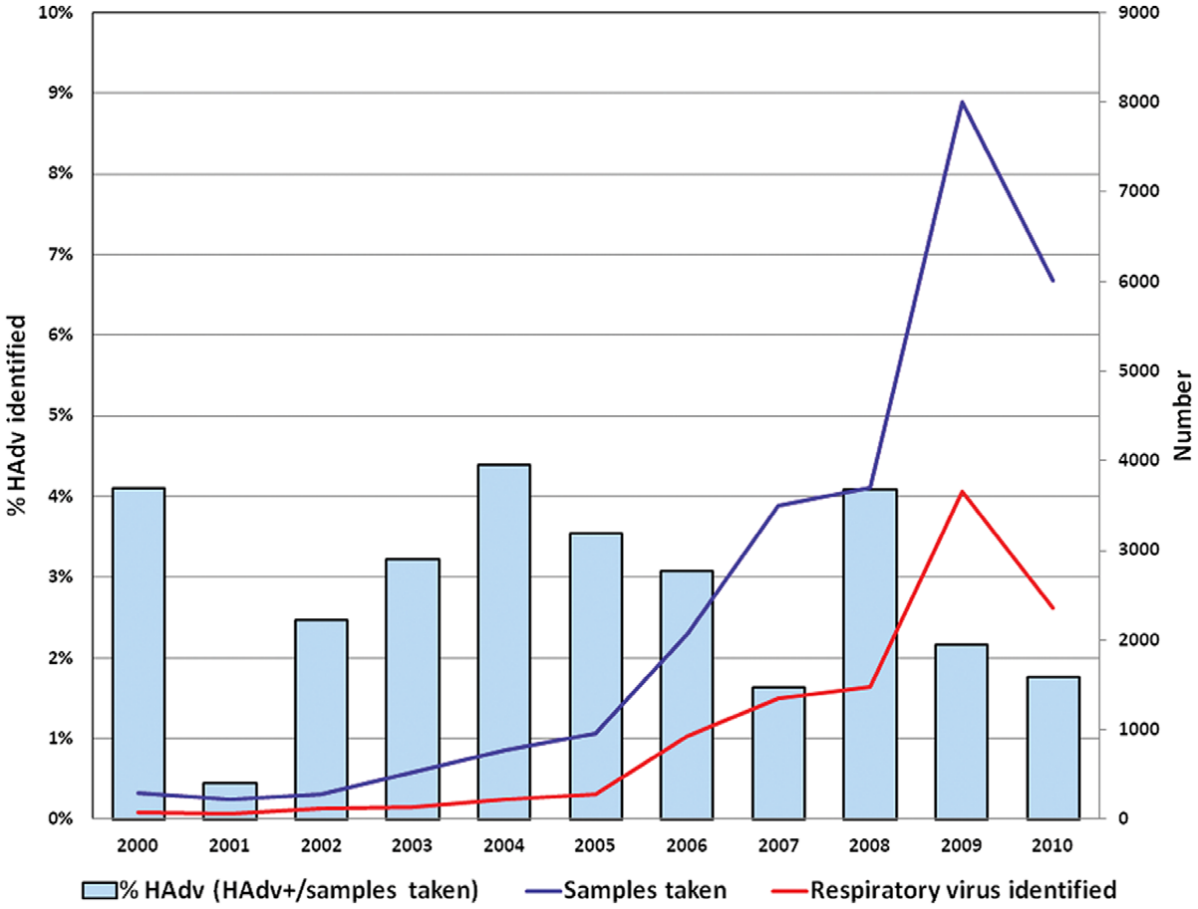
Samples collected, samples with a respiratory virus identified, and proportion of samples with HAdv identified by year. Peru, 2000–2010.

This study was conducted in 38 hospitals and health centers in 14 cities located in 11 provinces. Health facilities were located in northern coast provinces (Tumbes, Piura, and La Libertad), southern highlands provinces (Arequipa, Cusco, and Puno), jungle region provinces (Loreto, Madre de Dios, Junin, and Ucayali) and central coast (Lima). Sentinel surveillance was initiated in 2000 in Cusco, Lima, Loreto, and Piura. In 2006, to strengthen the national surveillance program, the Ministry of Health (MoH) of Peru invited NAMRU-6 to assist in increasing surveillance coverage by establishing the remaining sentinel sites in Tumbes, La Libertad, Arequipa, Puno, Madre de Dios, Junin, and Ucayali [26].

**Table 1:** Demographic characteristics of participants.

Characteristic	Samples taken (n = 26375)	Respiratory virus identified (n = 10647)	Other respiratory virus (n = 9991)	HAdv identified (n = 656)
**Sex**				
Male, n (%)	13768 (52.2)	5675 (41.2)	5302 (38.5)	373 (2.7)
Female, n (%)	12578 (47.7)	4972 (39.5)	4689 (37.3)	283 (2.3)
Missing, n	29	–	–	–
**Age (y)**				
Median (Q_25_–Q_75_)	12 (3–26)	10 (3–22)	11 (4–23)	3 (1–7)
Mode	1	1	1	1
Mean (SD)	17.4 (17.4)	15.5 (15.4)	16.1 (15.5)	6.6 (10.9)
**Age group**				
0–4, n (%)	8003	3150 (39.4)	2726 (34.1)	424 (5.3)
5–14, n (%)	6231	3152 (50.6)	2995 (48.1)	157 (2.5)
15–29, n (%)	6544	2588 (39.5)	2546 (38.9)	42 (0.6)
30–44, n (%)	3032	1002 (33.1)	988 (32.6)	14 (0.5)
45–59, n (%)	1618	520 (32.1)	506 (31.3)	14 (0.8)
≥60, n (%)	812	198 (24.4)	194 (23.9)	4 (0.5)
Missing, n	135	37	36	1
**Region of origin**				
Northern coast, n (%)	10362	4533 (43.7)	4143 (40.0)	390 (3.7)
Central coast, n (%)	3709	1253 (33.8)	1179 (31.8)	74 (2.0)
Southern highland, n (%)	3524	1431 (40.6)	1393 (39.5)	38 (1.1)
Jungle, n (%)	8749	3429 (39.2)	3275 (37.4)	154 (1.8)
Missing, n	31	1	1	–

Peru, 2000–2010.

* Other respiratory virus: Seasonal influenza A virus, influenza A (H1N1) pdm09 virus, influenza B virus, parainfluenza virus, respiratory syncytial virus, enterovirus, human bocavirus, human metapneumovirus, herpes simplex virus.

### Sample Collection

An oropharyngeal swab was obtained from study participants using flocked swabs and placed in Universal Transport Media (Diagnostic Hybrids, Quidel Corporation) and stored at –70°C until delivery on dry ice to NAMRU-6 in Lima, Peru (January 2006 and after), or to the Air Force Institute for Operational Health (AFIOH) Laboratory in San Antonio, Texas (March 2007 and before).

**Figure 3:**
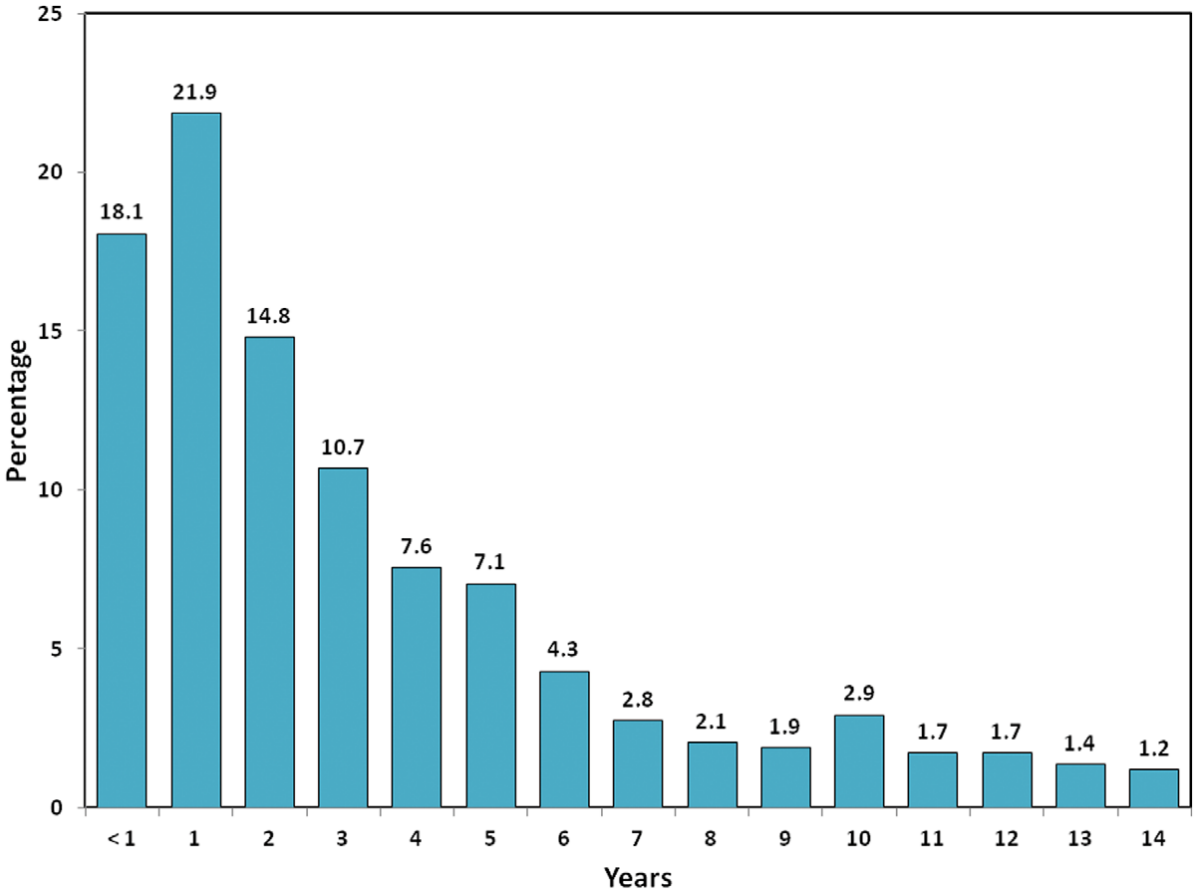
Age distribution of 581 children younger than 15 years diagnosed with HAdv infection. Peru, 2000–2010.

**Table 2:** Distribution of co-infections.

	N°	%
**Dual infection**		
Adenovirus – Seasonal influenza A	30	29.4
Adenovirus – Influenza A (H1N1) pdm09	18	17.6
Adenovirus – HSV*	15	14.7
Adenovirus – Parainfluenza	10	9.8
Adenovirus – Enterovirus	9	8.8
Adenovirus – Influenza B	7	6.9
Adenovirus – Human Bocavirus	5	4.9
Adenovirus – hMPV †	3	2.9
Adenovirus – Respiratory syncytial virus	2	2.0
**Triple infection**		
Adenovirus – Influenza B – HSV*	2	2.0
Adenovirus – Parainfluenza – HSV*	1	1.0
**Total**	102	100.0

Peru, 2000–2010.

* Herpes simplex virus.

† Human metapneumovirus.

### Virus Isolation and Identification

Isolation was performed at one of two laboratories. At AFIOH, samples were processed by the shell vial method [30] using two cell lines: primary monkey kidney (PMK) from Viro-Med, Minneapolis, MN; BioWhittaker, Walkersville, MD, and human alveolar adenocarcinoma (A549) purchased from Diagnostic Hybrids, Inc. (Athens, OH). Isolation was performed at NAMRU-6 by modified shell vial culture [31] using three commercial cell lines from the American Type Culture Collection (ATCC): Madin-Darby canine kidney (MDCK) obtained from ATCC® CCL-34™, rhesus monkey kidney (LLCMK2) cells obtained from ATCC® CCL-7™ [32], and African green monkey kidney (Vero 76) obtained from ATCC® CRL-1587™. Vero 76 was replaced with a Vero E6 cell line in 2007 obtained from ATCC® CRL-1586™. During the transition period from AFIOH to NAMRU-6, from January 2006 through March 2007, both laboratories analyzed the same samples (n = 2222) with good concordance to detect HAdv (Kappa = 0.83) [33].

**Table 3:** Clinical findings of patients with HAdv infection compared with other respiratory virus infections*.

Characteristic	HAdv	Other respiratory virus*	p value
Cough, n/N (%)	463/547 (84.6)	8991/9643 (93.2)	<0.001
Rhinorrhea, n/N (%)	429/531 (80.8)	8024/9499 (84.5)	0.023
Malaise, n/N (%)	435/545 (79.8)	8351/9638 (86.6)	<0.001
Sore throat, n/N (%)	351/542 (64.8)	7372/9513 (77.5)	<0.001
Headache, n/N (%)	286/542 (52.8)	6950/9605 (72.4)	<0.001
Conjunctival injection, n/N (%)	269/529 (50.9)	4421/9363 (47.2)	0.104
Muscle soreness, n/N (%)	163/537 (30.4)	4666/9521 (49.0)	<0.001
Eye pain, n/N (%)	100/524 (19.1)	3200/9393 (34.1)	<0.001
Rhonchi, n/N (%)	67/527 (12.7)	1013/9340 (10.8)	0.182
Photophobia, n/N (%)	32/522 (6.1)	1017/9329 (10.9)	0.001
Dizziness, n/N (%)	25/524 (4.8)	1415/9333 (15.2)	<0.001
Earache, n/N (%)	24/525 (4.6)	917/9340 (9.8)	<0.001
Wheezing, n/N (%)	22/523 (4.2)	630/9331 (6.8)	0.023

Peru, 2000–2010.

* Seasonal influenza A, Influenza A (H1N1) pdm09, HSV, influenza B, parainfluenza, enterovirus, HBoV, hMPV and respiratory syncytial virus. Those with co-infections or with missing responses were removed from the analysis.

Each cell line was prepared in 24-well tissue culture plates**.** The growth medium for the three cell lines consisted of Eagle’s minimum essential medium (Quality Biological, Cat.112-018-131) with 10% fetal bovine serum (F-4135, Sigma) and Antibiotic Antimycotic Solution (10,000 units penicillin, 10 mg streptomycin, and 25 µg amphotericin B; A-5955, Sigma).

**Figure 4:**
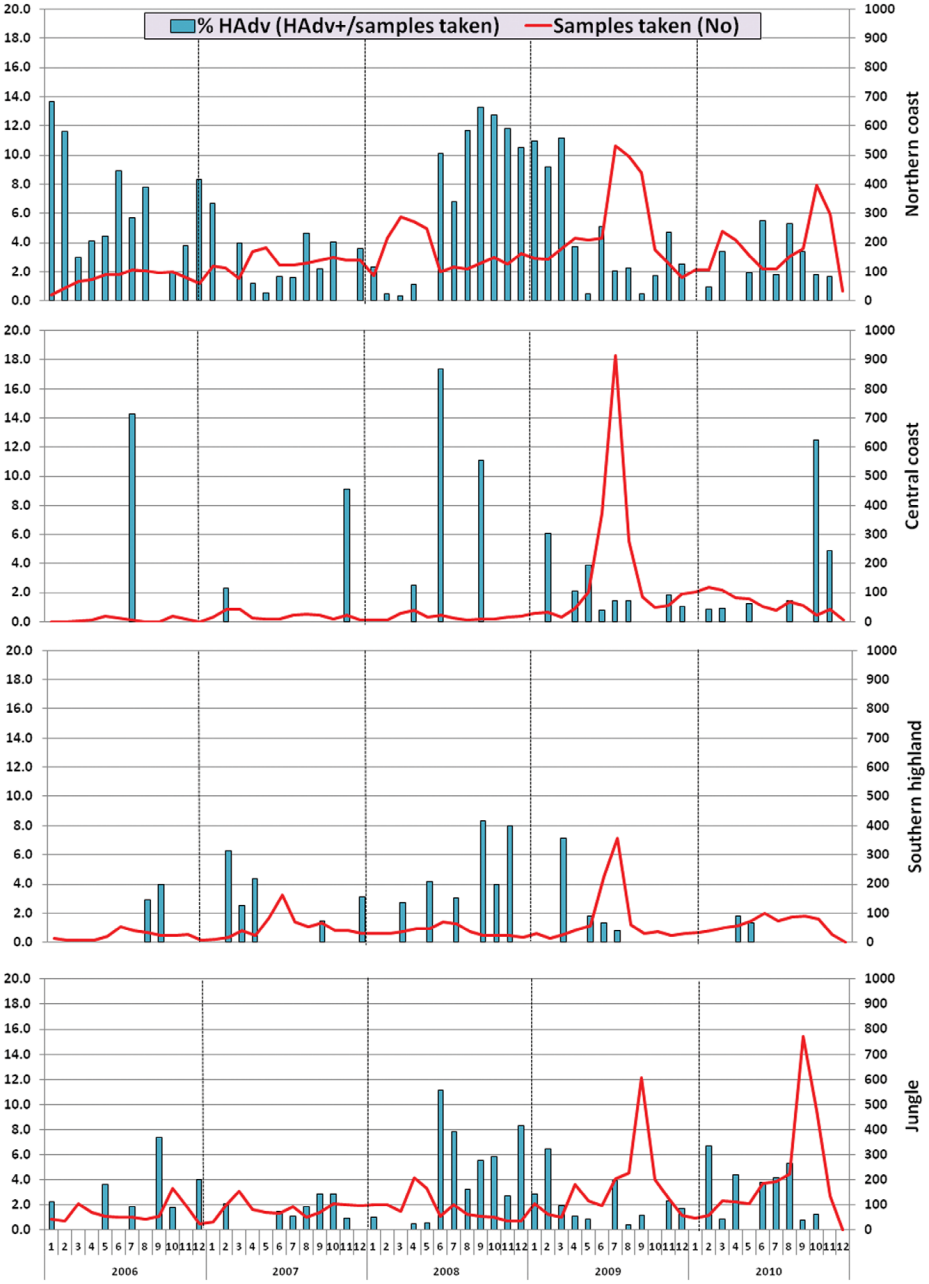
Distribution of HAdv isolates per month and year by regions. Peru, 2006–2010. Northern coast: Tumbes, Piura and La Libertad; Central coast: Lima; Southern highland: Arequipa, Cusco and Puno; Jungle: Loreto, Madre de Dios, Junin and Ucayali.

Upon the appearance of a cytopathic effect (CPE) or after ten (or 13 in the case of Vero cells) days of culture, the cells were spotted onto microscope slides. Cell suspensions were dried and fixed in chilled acetone for 30 minutes. The Respiratory Virus Screening and Identification Kit (D3 DFA Respiratory Virus Diagnostic Hybrids, Athens, OH) was utilized for the identification of HAdv. All assays were performed following the manufacturer’s established protocols.

**Table 4:** Distribution of genotyped samples per collection year, region, age group, gender, and type of infection.

	Subgroup			Total HAdv subtyped (n = 226) n (%)	Total HAdv specimens (n = 551)
	B (n = 39)	C (n = 171)	E (n = 16)		
**Collection year**					
2006	13	31	0	44 (68.8) [28]	64
2007	5	24	0	29 (50.9) [28]	57
2008	12	65	8	85 (56.3) [28]	151
2009	6	26	8	40 (23.1)	173
2010	3	25	0	28 (26.4)	106
**Collection sites**					
Northern coast	28	119	12	159 (46.4)	343
Central coast	1	13	1	15 (31.3)	48
Southern highland	3	8	0	11 (39.3)	28
Jungle	7	31	3	41 (31.1)	132
**Patient age group**					
0–4	20	128	6	154 (43.4)	355
5–14	16	27	8	51 (39.8)	128
15–29	1	10	1	12 (30.8)	39
30–44	0	4	0	4 (30.8)	13
45–59	1	2	1	4 (33.3)	12
≥60	1	0	0	1 (25.0)	4
**Gender**					
Male	27	91	11	129 (41.7)	309
Female	12	80	5	97 (40.1)	242
**Type of infection**					
Single	36	156	5	197 (44.2)	446
Co-infection	3	15	11	29 (27.6)	105

Peru, 2006–2010.

**Figure 5:**
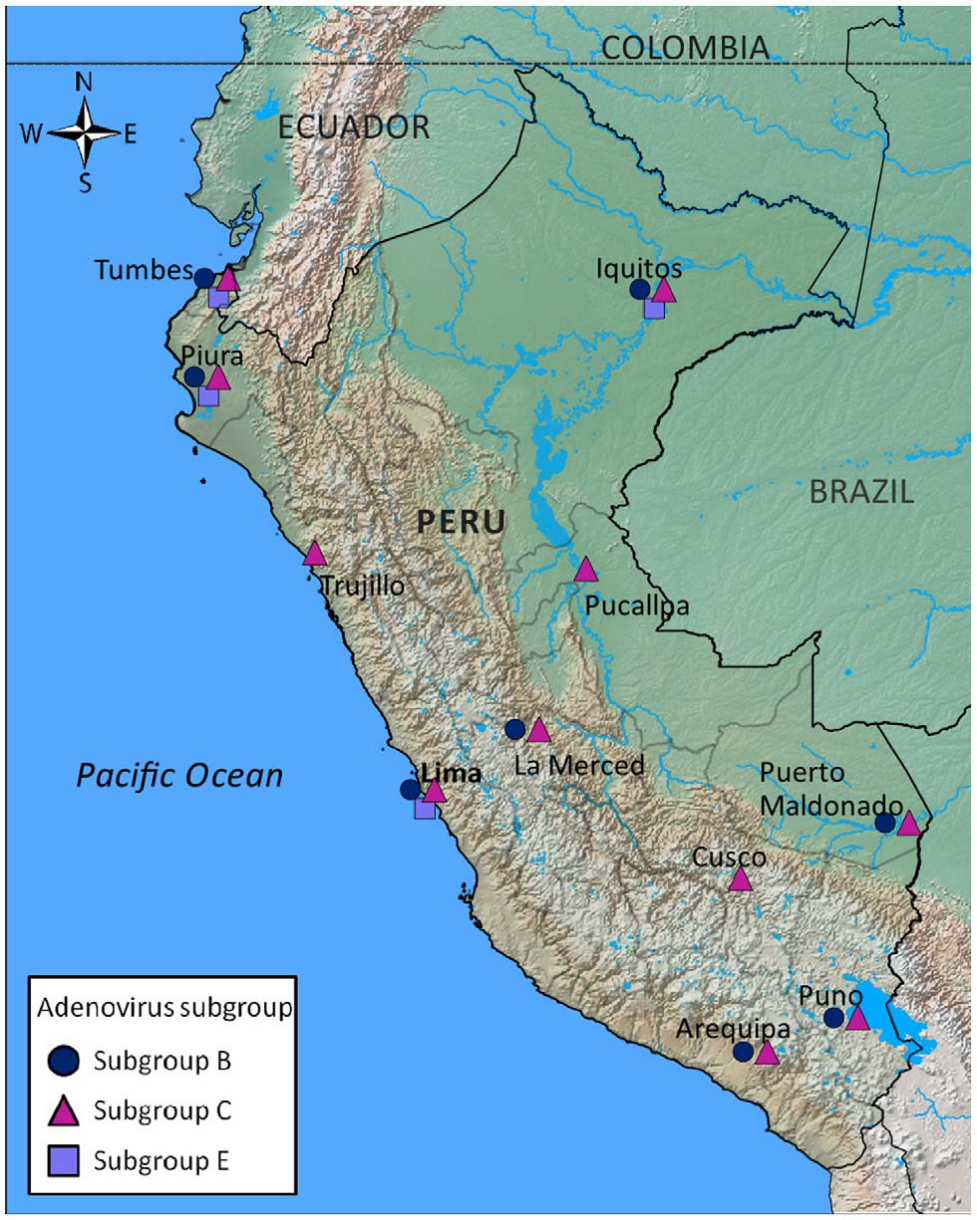
Map of Peru with the distribution of the HAdv subgroups among the 11 sentinel surveillance provinces encompassing 14 cities and 38 health centers. The provinces were: Tumbes (Tumbes: 3 sites); Piura (Piura: 2 sites, Sullana: 3 sites); La Libertad (Trujillo: 2 sites); Lima (Lima: 6 sites); Arequipa (Arequipa: 1 site); Puno (Puno: 1 site, Juliaca: 1 site); Cusco (Cusco: 1 site); Madre de Dios (Puerto Maldonado: 3 sites); Junin (La Merced: 1 site); Ucayali (Pucallpa: 1 site); Loreto (Iquitos: 12 sites, Yurimaguas: 1 site). Peru, 2006–2010.

**Figure 6:**
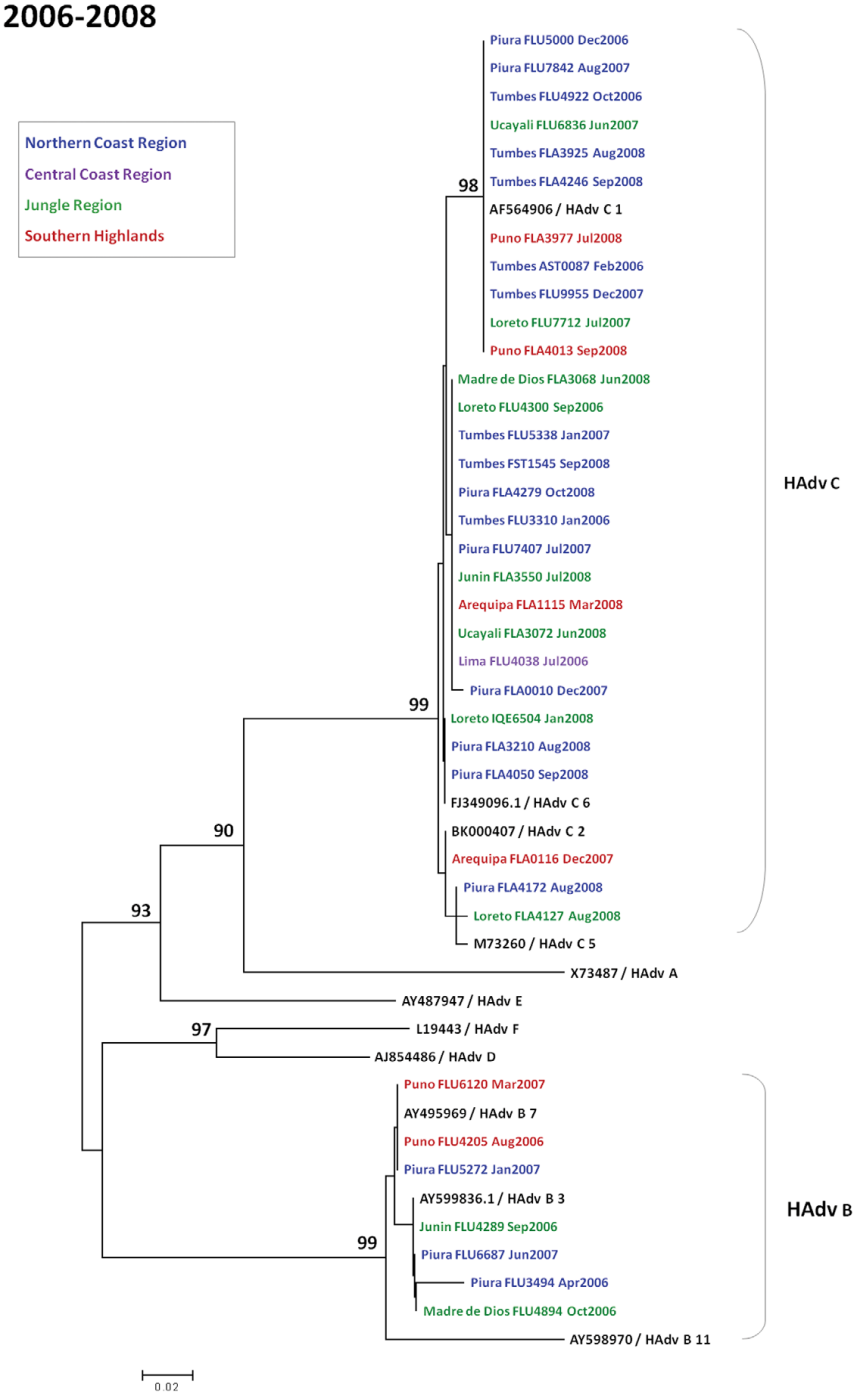
Phylogenetic tree of adenovirus isolates in Peru during 2006–2008. The last 280 nucleotides of the adenovirus hexon gene were amplified, sequenced, and compared to published sequences from GenBank. Samples are labeled according to the following format: “Province of collection - Sample Code - Month- Year of collection.” The comparison sequences are complete genome sequences from GenBank and are presented in the following format: “Accession Number/Serotype in Bold.” Geographical regions are color coded: northern coast region (blue), central coast region (magenta), jungle region (green), and southern highlands region (red). Nucleotide sequences were aligned using Clustal X. Phylogenetic analyses were performed using the Kimura two-parameter model as a model of nucleotide substitution and using the neighbor-joining method to reconstruct phylogenetic trees (MEGA version 2.1). The samples are grouped into species.

**Figure 7:**
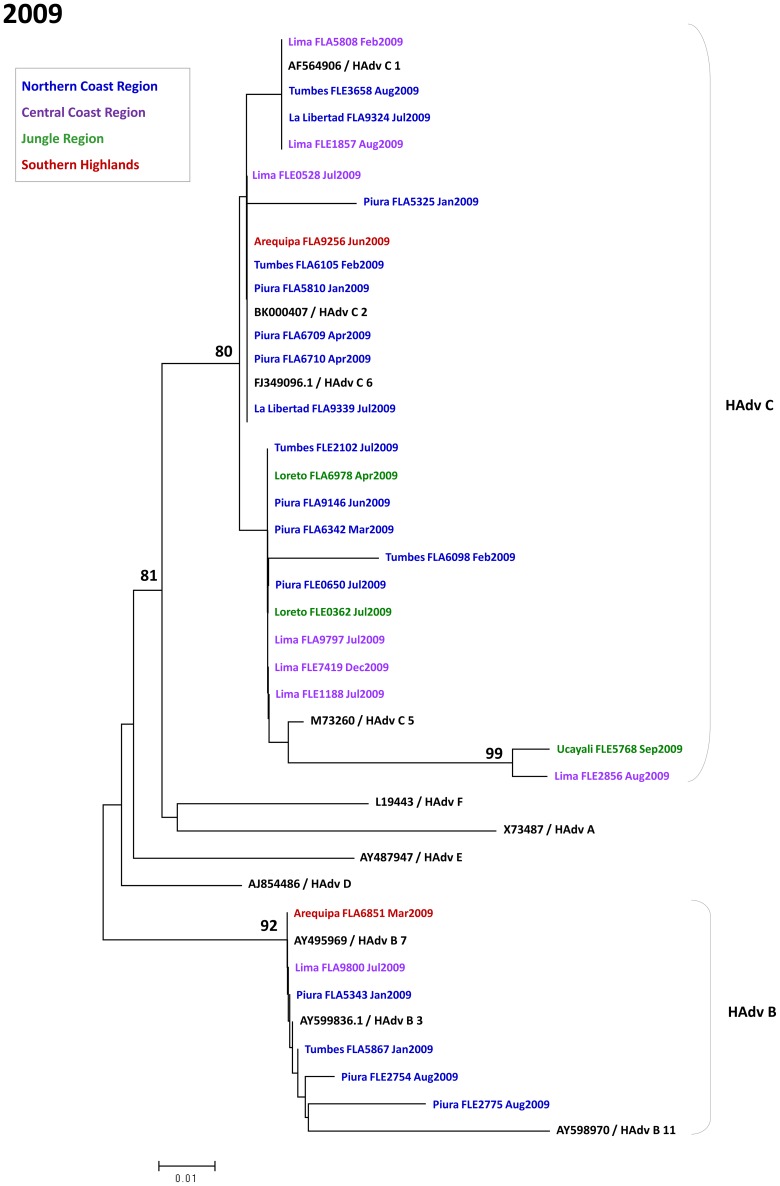
Phylogenetic tree of adenovirus isolates in Peru during 2009.

**Figure 8:**
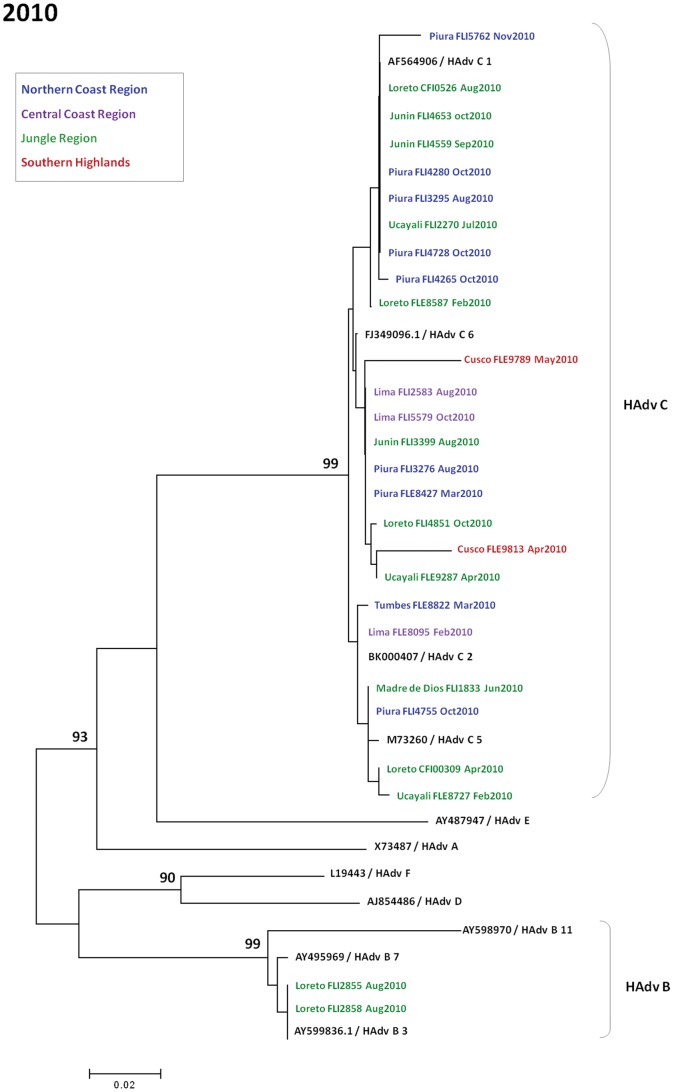
Phylogenetic tree of adenovirus isolates in Peru during 2010.

### PCR and Sequencing

Using a random number generator on MS Excel, 226 HAdv samples were selected for further characterization using PCR and sequencing. Viral DNA was extracted from immunofluorescence-positive samples using the AmpliTaq Gold® kit (Applied Biosystems; CA) and tested by polymerase chain reaction (PCR). The last 280 base pairs of the HAdv hexon gene were amplified as previously described [28] with the following specific primers: Adeno 3 (5′-CCTTTGGCGCATCCCATTCT-3′) and Adeno 4 (5′-TGGGCACCTATGACAAGCGC-3′). For direct sequencing, gene fragments were amplified and sequenced with the use of the Big Dye terminator cycle sequencing kit (version 3.1, Applied Biosystems, Ann Arbor, MI) on a Genetic Analyzer system (version 3130×L, Applied Biosystems, Ann Arbor, MI). Gene sequences were assembled, aligned, and edited using Sequencer and BioEdit software (version 7.0.0, Isis Pharmaceuticals, Inc.; Dublin, Ireland). Phylogenetic trees were generated with CLUSTAL X version 2.0.1 and MEGA version 3.1 software.

### Ethics

These protocols were approved as less than minimal risk research by the Naval Medical Research Center (NMRC), Silver Spring, MD. Institutional Review Board (IRB; NMRCD.2000.0006, NMRCD.2002.0019), and NAMRU-6 (PJT.NMRCD.085) following the regulations of human subjects research (45 CFR 46) from the U.S. Department of Health and Human Services. Authorization was given to perform the study using an information sheet approved and stamped by the IRB, as this was part of clinical care and routine surveillance of patients with upper respiratory infections from the Peruvian MoH without any additional intervention, verbal consent was obtained from all participants after the contents of the information sheet were explained; verbal assent from children 8–17 years old was obtained in addition to their parents’ approval; an information sheet copy was provided to each study subject. This method of consent was accepted by the NMRC and NAMRU-6 IRB, as well as the Peruvian MoH.

### Statistical Analysis

The clinical-epidemiological forms were entered into a database created in Microsoft Office Access 2003. Proportions were compared using the Pearson Chi-Square and Fisher exact tests. Continuous variables with a normal distribution were compared using the independent samples t-test; otherwise, the Kruskal-Wallis test was applied. For all confidence intervals (CI) or statistical tests, the level of confidence was 95%. A two-tailed critical value of alpha = 0.05 was used for all statistical analyses using SPSS Statistics software version 17.0 (SPSS Inc.; Chicago, IL). Variables with data missing on 10% or more of the forms were not analyzed. The analyses of geographic distribution, seasonality, and sequencing were conducted only on samples from years 2006 to 2010, the period of time when all the sites were participating in sentinel surveillance.

## Results

### General Findings

From January 2000 through December 2010, 26,375 participants were enrolled (Figure 1); one or more respiratory viruses were found in 10,647 (40.4%) of participants. Adenoviral infections represented 6.2% of viral infections (656 HAdv identified/10,647) and 2.5% of all cases of ILI (656/26,375).

Figure 2 shows the number of samples obtained, the number of samples with any respiratory virus identified, and the proportion of HAdv isolations per enrollment year (HAdv positive samples/number of samples taken). The number of samples collected increased over the years, mainly after 2006 when all the sites were participating in the network of the MoH [26]. The HAdv isolation rate varied through the years, ranging from 0.4 to 4.4%. In 2009, the number of samples obtained rose considerably (n = 7998) secondary to increased surveillance due to the presence of the pandemic influenza A/H1N1 virus as previously described by our group [27].

Table 1 shows demographic features of the study population. The male/female (M/F) ratio for HAdv infection was 1.3 (*X* ^2^ = 5.7, *p* = 0.017); for other respiratory virus infections this was 1.1 (*X* ^2^ = 4.2, *p* = 0.040). The mean age for those with HAdv and other respiratory virus infections was 6.6 and 16.1 years, respectively (t = 20.9, p<0.001). Compared to the identification of other viruses, the percentage of HAdv isolated decreased as age increased. In children younger than five years, the percentage of HAdv found was 5.3% and the lowest percentage, 0.5%, occurred among participants between 30 and 44 years old and ≥60 years old.

The proportion of HAdv cases (number of positive HAdv/total number of samples) was highest in the sites of the northern coast (3.8%), followed by the samples obtained from sites of the central coast (2.0%) and the jungle region (1.8%) (*X* ^2^ = 121.1, p<0.001); this fact was independent of year of enrollment and number of samples obtained in each region.

Among children younger than 15 years old (n = 581), the largest proportion of HAdv cases occurred in participants of one year of age (21.9%), followed by those younger than one year of age (18.1%). For those less than 15 years of age, every age above three accounted for less than 10% of the total HAdv cases (Figure 3); comparison with other respiratory viruses can be found in Figures S1 and S2.

One or more co-infecting viruses were detected in 15.5% (n = 102) of HAdv-infected participants. Dual viral infection was detected in 99 participants, and triple infection was detected in 3 (Table 2). The most common co-infection was HAdv-seasonal influenza A (H1N1, H3N2), following by HAdv-pandemic influenza A (influenza A (H1N1) pdm09) and HAdv-herpes simplex virus (HSV).

The median duration of illness at the time of study enrollment among HAdv-infected participants was 2 days (Q_25_–Q_75_: 1–3). The median axillary temperature recorded was 38.0°C (Q_25_–Q_75_: 37.6–38.7). Cough and sore throat, two inclusion criteria, were found in 84.6% and 64.8%, respectively. The two other most common symptoms at the time of examination in patients with single infection with HAdv were rhinorrhea (80.8%) and malaise (79.8%). Headache and conjunctival injection were also common and affected more than half of HAdv-infected participants (Table 3).

Participants infected with non-HAdv respiratory viruses possessed more varied clinical findings compared with those infected only with HAdv (Table 3). Cough, malaise, sore throat, headache, muscle soreness, eye pain, photophobia, and dizziness occurred more often in those infected with a virus other than HAdv when compared to those infected with HAdv. On the other hand, conjunctival injection and rhonchi were present more often among those infected with HAdv, although these were not statistically significant (p>0.05).

### Seasonality

Figure 4 shows the spatio-temporal distribution of HAdv cases in Peru from 2006 to 2010. HAdv was detected throughout the year. Geographically, the proportion of HAdv infection (number of positive HAdv cases/number of samples taken) was more common in sites of the northern coast regions and the jungle than in the central coast or highland sites.

In June 2008, the proportion of HAdv infections increased in sites of the northern coast, central coast, and the jungle region to 10%, 17%, and 11%, respectively. This high proportion of HAdv remained in the northern coast up to March 2009, ranging between 7% and 13%.

### Genotyping

The hexon gene from 226 randomly selected HAdv isolates (41% of all) obtained from 2006 to 2010 was genotyped; genetic analysis of part of this sample set has been discussed previously [26], [28]. The variable region of the hexon gene was chosen for sequencing to accurately detect different subgroups of HAdv and also to be consistent with previous publications [34]. The characteristics of these samples are shown in Table 4. From 2006 to 2010, the predominant subgroup was subgroup C (75.7%), followed by subgroup B (17.3%). Subgroup E circulated between July 2008 and April 2009, with the first case from the jungle region, and later cases from the northern and central coast regions. No differences in age or gender were found among the different subgroups. The geographical distribution of the different subgroups is displayed in Figure 5.

Figures 6, 7, and 8 show phylogenetic trees of sequenced HAdv sub-divided between 2006–2008 [28], 2009, and 2010. Within subgroup C, subtypes observed were C1, C2, C5, and C6. Within subgroup B, subtypes 3 and 7 were seen. Relative uniformity was observed among genotypes in the five years of analysis.

## Discussion

Although HAdv is able to produce a broad range of infections in humans, little has been published about it in Peru. We had the unique opportunity to sample Peruvian patients with ILI or SARI presenting in various regions of the country over many years and found that HAdv was present in 2.5%. Oftentimes, prevalence of this pathogen is underestimated due to the lack of viral isolation capability in most healthcare settings and the mild disease that HAdv often causes. Our surveillance system addressed both of these deficiencies by performing isolation on two to three different cell lines for every respiratory sample obtained and by including mainly outpatient clinic settings, which were more likely to encounter mild disease than inpatient wards. However, our outpatient focus often resulted in a lack of follow-up of patients severe enough to be hospitalized, limiting our ability to describe severe complications as other groups have [3], [5], [6], [7], [8].

In South America, only a handful of similar studies have described the clinical and epidemiological findings associated with HAdv infection. Although inclusion criteria, laboratory methods, and time periods studied were not always identical between studies, comparisons are still possible. Using isolation as the goal standard, a Brazilian research group found HAdv in 2.6% of adults with ILI, accounting for 1.5% in military members and 3.4% in civilians between 2000 to 2002 [35]. On the other hand, researchers from Zulia, Venezuela, who also relied on viral isolation in participants of any age reported HAdv in 12.7% of patients with ILI or SARI during 2005 and 2006 [36], five times higher than what we found. This difference is probably because the Venezuelan study included more inpatients, which tend to have more severe disease, than our study. Although HAdv may cause mild disease, it also has the potential for severe outcomes, as others have found a high proportion of HAdv infection in those with severe disease [37].

While children under 15 years of age comprised 54.0% of the participants recruited in our respiratory surveillance network, they accounted for 88.6% of HAdv cases identified. We also found that 5.3% of children younger than 5 years with ILI or SARI were infected with HAdv, a finding similar to many other South American HAdv studies. In Colombia, 2.6% (47/1743) of respiratory samples from children under 5 with ILI or SARI contained HAdv [38]. In Brazil, samples collected from pediatric patients with ILI or SARI (<5 years old) demonstrated a broad range of proportions containing HAdv: 6.8% (23/336) in São Paulo [39], 6.0% (52/862) in Rio Grande do Sul [40], and 2.3% (11/482) in Salvador, Bahia [41]. Among samples only from children with SARI, proportions of HAdv ranged between: 2.8% (28/1002) from 1984 to 1987 [42] and 1.9% (916/48544) from 1999 to 2010 [43] in Argentina; 9.3% (457/4927) from 1989 to 2001 in Chile [44], and 7.0% (9/128) from 1996 to 1997 [45] and 6.2% (2/32) from 2006 to 2007 [46] in Cuba.

The M/F ratio found in our population was 1.3, an interesting finding noted by others. Studies from Colombia, Chile, Argentina, Brazil, and Cuba found a significantly higher M/F ratio, 2.3 [38], 1.5 [44] and 2.2 [47], 1.8 [48], 1.5 [49], and 2.0 [45], respectively. Other authors in USA, Spain, Taiwan, and Israel also found high M/F ratios, 1.4 [50], 1.5 [25], 1.6 [51], and 1.5 [52], respectively. The reasons for this discrepancy have not yet been elucidated.

We demonstrated that 15% of all patients with ILI and HAdv isolated also had another respiratory virus identified. This was the case despite relying on insensitive methods to detect RSV and only investigating for HBoV in the final two years of the study. We did not note a higher prevalence of any clinical manifestations upon comparing the co-infected patients versus those with just HAdv detected. Similarly, a study from Chile found no difference in clinical severity between children co-infected with RSV-HAdv compared with children with HAdv monoinfection [44]. It has been reported that 35% of asymptomatic children may have respiratory viruses, with HAdv being the most frequent, followed by rhinovirus and human metapneumovirus [53]. This fact could explain the presence of two or more viruses in our ILI patients. The presence of these viruses could enhance the potency of a subsequent respiratory viral infection with a different virus. Additional studies should be done to determine the role of each one of these viruses found in patients with ILI.

Compared with those infected with other respiratory viruses, we found no statistically significant higher prevalence of any clinical manifestation in those infected with HAdv. Although not statistically significant, conjunctival injection appeared to affect those with HAdv more often than those with another respiratory virus, not surprising since conjunctival pathology has been well-described with HAdv infection [7], [15], [16], [20], [21].

Over the five years of surveillance, we noted no seasonality with HAdv infection. This lack of seasonality of HAdv infection has been described in other South American countries such as Colombia [38], Chile [44], and Brazil [54]. Nevertheless, we noted certain periods, such as the latter half of 2008, when either the absolute number or the proportion of HAdv identified (per total number of ILI cases) were elevated. The latter half of 2009 also was significant for an increase in the number of isolates of HAdv collected. This was due to increased surveillance for pandemic influenza [27], and therefore, no concomitant increase in the proportion of HAdv cases (per total number of ILI cases) was noted. These trends are best illustrated in the northern coastal region, where not only more HAdv isolates were consistently collected, but also a larger proportion compared to total ILI cases during the reporting period. No obvious reason is evident for this regional predilection, but it might be related to the city’s year-round warm and dry climate, conditions that have been correlated with increased HAdv detection rates by others [55].

By genotyping 41% of the HAdvs collected, it was possible to depict HAdv phylogeny throughout Peru. In all four regions (northern coast, central coast, southern highlands, and jungle) subgroup C predominated, subgroup B was the second most common, and subgroup E was the least common (except for the central coast where B and E were identified in the same number). Likewise, the bordering country of Brazil [41], [56], as well as the non-bordering Latin American countries of Mexico [57] and Cuba [45], also observed a predominance of subgroup C. On the other hand, two other countries bordering Peru, Colombia [38], [58] and Argentina [43], noted a predominance of subgroup B.

Although this passive surveillance network allowed for the collection of a large amount of samples from throughout the country, the one-point-in-time nature of our data and specimen collection limited the ability to make conclusions about disease evolution or severity of illness. Another limitation, the unequal distribution and participation level of the sites in our country-wide respiratory surveillance network, prevented the ability to make broad temporal-spatial generalizations regarding HAdv. Nevertheless, we were able to demonstrate the clinical manifestations of HAdv infection in the initial patient visit, the phylogenetic distribution of HAdv throughout the country and over time, and that HAdv circulates in Peru throughout the year, all items that contribute to the relatively sparse description of HAdv disease in South America. Future longitudinal studies will help better characterize the clinical course of patients with HAdv in Peru, as well as the role of co-infections in the evolution of illness.

## Supporting Information

Figure S1 **Age distribution of children younger than 15 years diagnosed with HAdv, seasonal influenza A virus, influenza B virus, influenza A (H1N1) pdm09 virus, parainfluenza virus, and respiratory syncytial virus (RSV) infections.** Peru, 2000–2010.
(TIF)

Figure S2 **Box-and-whisker plot of age (years) around the median age (horizontal line) for each of the viruses isolated.** Only participants with one virus detected were evaluated in this analysis. Boxes extend from 25–75th percentiles. Whiskers extend to the largest and smallest observed values in the distribution which fall within 1.5 times the box length around the median. Circles represent outlier ages.
(TIF)
