# Supplementary figures and images for: Adenovirus Respiratory Tract Infections in Peru
Source: PLoS One. 2012 Oct 8;7(10):e46898. doi: 10.1371/journal.pone.0046898 (PMC3466214; doi:10.1371/journal.pone.0046898)

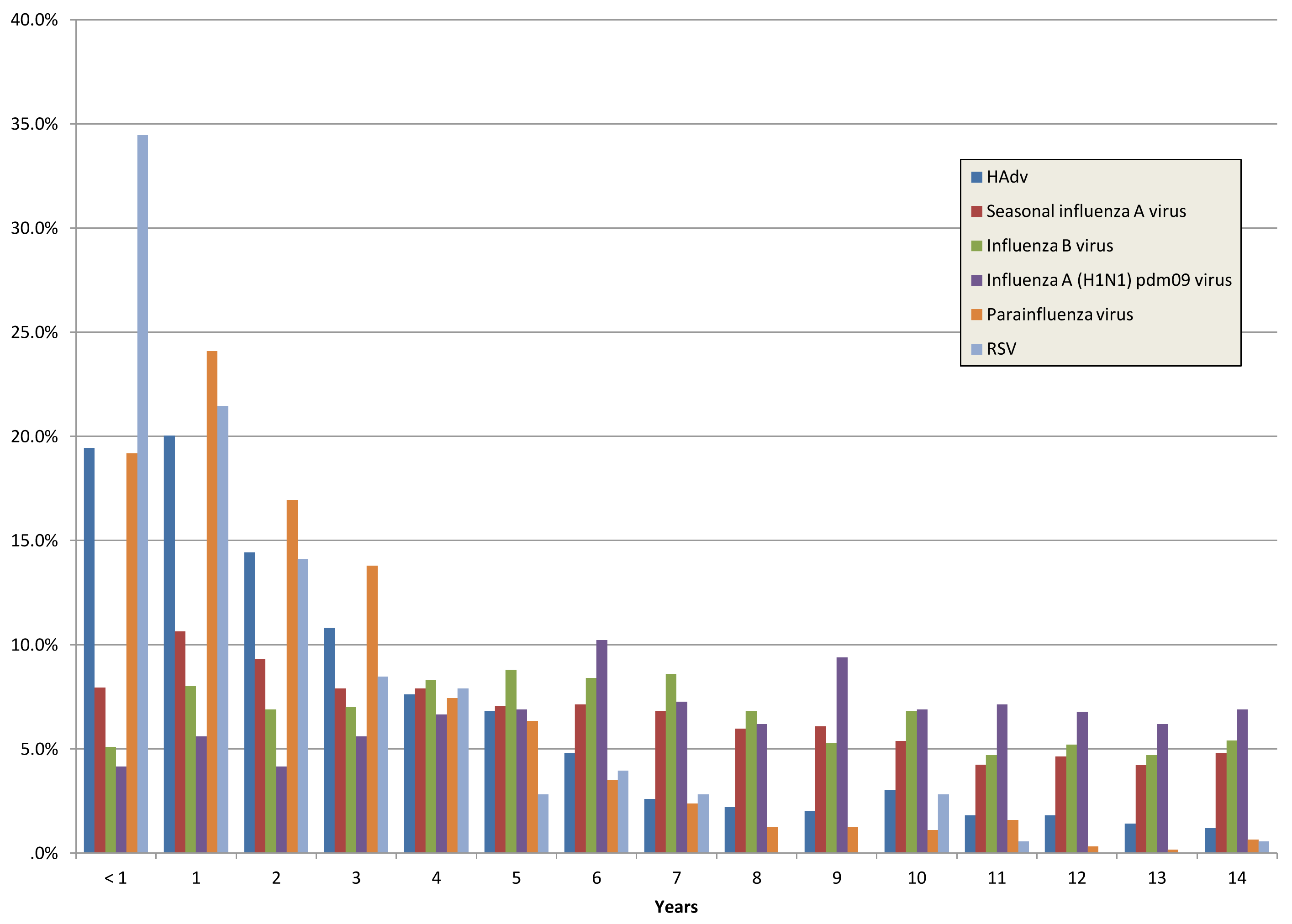

Supplement: Figure S1 — Age distribution of children younger than 15 years diagnosed with HAdv, seasonal influenza A virus, influenza B virus, influenza A (H1N1) pdm09 virus, parainfluenza virus, and respiratory syncytial virus (RSV) infections. Peru, 2000–2010. (TIF) [file pone.0046898.s001.tif]

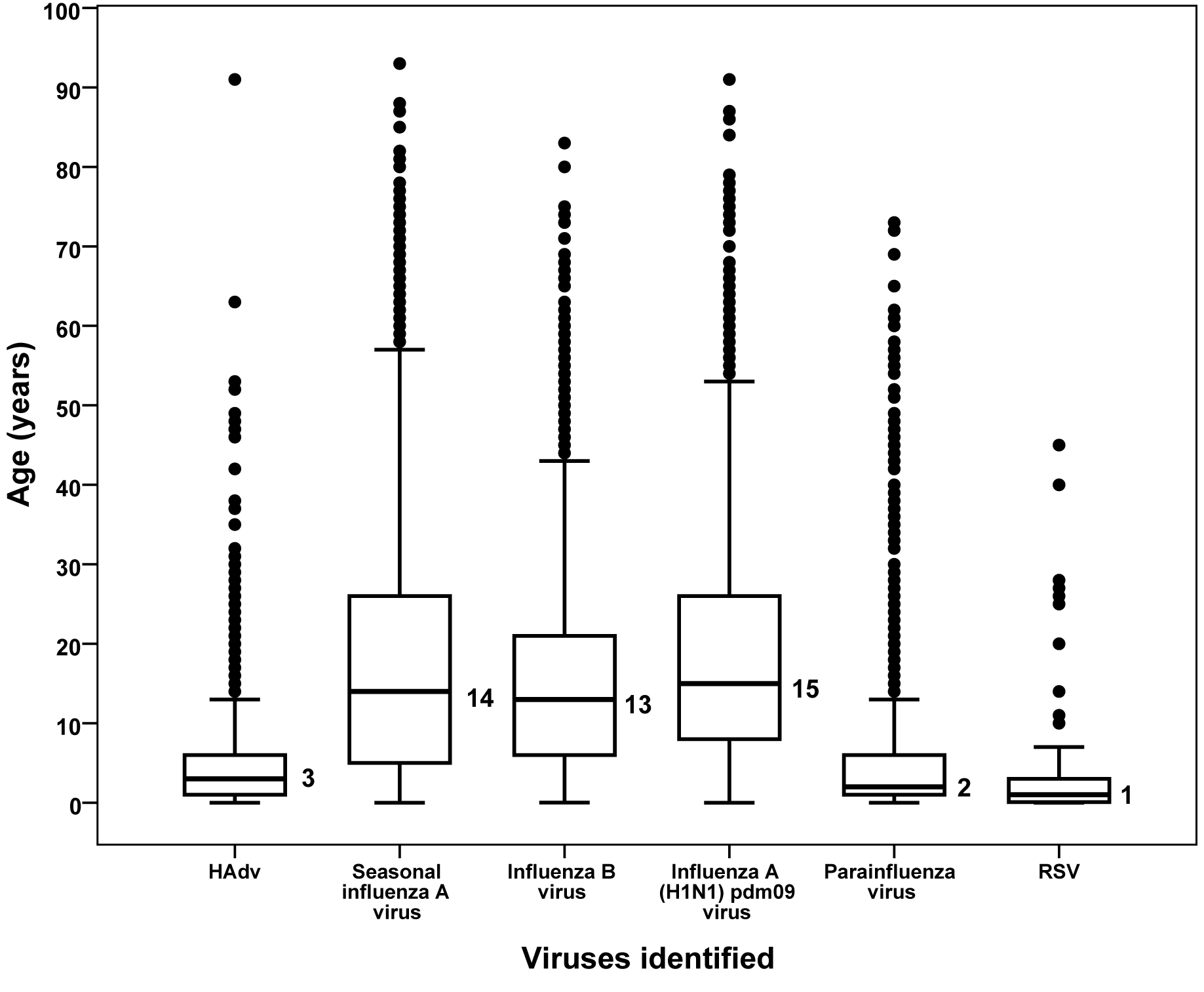

Supplement: Figure S2 — Box-and-whisker plot of age (years) around the median age (horizontal line) for each of the viruses isolated. Only participants with one virus detected were evaluated in this analysis. Boxes extend from 25–75th percentiles. Whiskers extend to the largest and smallest observed values in the distribution which fall within 1.5 times the box length around the median. Circles represent outlier ages. (TIF) [file pone.0046898.s002.tif]
